# Assessment of asymptomatic *Leishmania* infection in people living with HIV: a long-term follow-up study in Northeastern Brazil

**DOI:** 10.3389/fmed.2025.1682423

**Published:** 2025-10-27

**Authors:** Matheus Henrique Gonçalves Aguiar, Diego Lins Guedes, Walter Lins Barbosa Júnior, Elis Dionísio da Silva, Gilberto Silva Nunes Bezerra, Cristine Vieira Bonfim, Amanda Virginia Batista Vieira, Bruna Eduarda Freitas Monteiro, Roberta dos Santos Souza, Lucyo Flávio Bezerra Diniz, Samuel Ricarte de Aquino, Luiz Gustavo Mendes, Alda Maria Justo, Zulma Maria de Medeiros

**Affiliations:** ^1^Postgraduate Program in Health Sciences, University of Pernambuco (Campus Santo Amaro), Recife, Brazil; ^2^Department of Life Sciences, Federal University of Pernambuco, Caruaru, Brazil; ^3^Department of Parasitology, Aggeu Magalhães Institute, Oswaldo Cruz Foundation (Fiocruz), Recife, Brazil; ^4^Department of Life & Health Sciences, Dundalk Institute of Technology, Dundalk, Ireland; ^5^Social Research Division, Joaquim Nabuco Foundation, Ministry of Education, Recife, Brazil; ^6^Postgraduate Program in Health Biosciences and Biotechnology, Aggeu Magalhães Institute, Oswaldo Cruz Foundation (Fiocruz), Recife, Brazil; ^7^University Hospital, Federal University of Vale do São Francisco, Petrolina, Brazil; ^8^Ministry Health, Petrolina, Brazil; ^9^Department of Nursing, University of Pernambuco (Campus Petrolina), Petrolina, Brazil

**Keywords:** visceral leishmaniasis, *Leishmania infantum*, longitudinal evaluation, HIV coinfection, disease progression

## Abstract

In people living with HIV (PLWH), surveillance for *Leishmania* infection is crucial for identify those at risk of developing visceral leishmaniasis (VL). *Leishmania*-HIV coinfection worsens patient outcomes and increases mortality and relapse rates. We conducted a prospective study (2017–2023) in Northeast Brazil to assess the long-term outcomes of asymptomatic *Leishmania*-HIV coinfected outpatients and *Leishmania-*negative PLWH. Participants were drawn from a cross-sectional study performed in 2017, which identified both *Leishmania-*HIV coinfected and *Leishmania-*negative PLWH. Epidemiological, clinical, and laboratory data were collected from medical records (2017–2023). In 2023, these individuals were retested for *Leishmania* using serological tests and conventional polymerase chain reaction (PCR). Categorical variables were compared using the chi-square test, and non-parametric tests were used for quantitative variables. During follow-up, six individuals developed VL: five from the coinfected group and one from the non-coinfected PLWH group (OR 10.4; 95% CI 1.2–94.2; *p* = 0.023). Three patients experienced relapse: one from the PLWH group and two from the coinfected group. There was one death in the *Leishmania-*HIV group. In 2023, 80 patients were retested; five coinfected patients remained positive for VL by one or more tests, and two PLWH patients seroconverted for VL. Our findings underscore the critical need for long-term follow-up of asymptomatic *Leishmania*-HIV patients to mitigate disease progression and associated complications.

## 1 Introduction

Visceral leishmaniasis (VL) is a serious systemic disease caused by protozoa of the *Leishmania donovani* complex. In Brazil, the causative parasite is *Leishmania infantum* (1, 2). Typical symptoms of VL include recurrent fever, weight loss, hepatosplenomegaly, and pancytopenia (3, 4). Pederiva et al. (5) demonstrated that asymptomatic *Leishmania* infection is frequent in VL-endemic regions, particularly in developing countries such as Brazil and India.

People living with HIV (PLWH) in VL-endemic areas have an elevated risk of *Leishmania* infection compared with HIV-negative individuals (6–8). In the Americas, Brazil accounts for 93% of all reported cases of *Leishmania*–HIV coinfection (9, 10). The majority of these cases are caused by *Leishmania infantum* in the country’s Northeast region (7, 11–16). HIV infection accelerates the clinical progression from asymptomatic *Leishmania* infection to symptomatic VL. In patients with advanced immunosuppression, the disease can present with more severe and atypical manifestations (17). These atypical manifestations may include hemorrhagic events (e.g., epistaxis, ecchymosis, and hematuria), as well as dyspnea, diarrhea, and the involvement of organs not typically affected by *Leishmani*a. Reported atypical sites include the skin, oral mucosa, larynx, lungs, pleura, esophagus, stomach, small intestine, pancreas, and kidneys (18).

Detection of asymptomatic *Leishmania*-HIV coinfection relies on serological, molecular, and parasitological tests (4, 5). The “gold standard” for VL diagnosis, however, is parasitological confirmation. In Brazil, highly sensitive parasitological diagnoses, such as spleen, liver, and bone marrow aspiration, are commonly employed (19). However, there is currently no gold-standard test or consensus definition for diagnosing asymptomatic *Leishmania* infection (20, 21). Therefore, optimal surveillance for PLWH in VL-endemic areas requires a combination of diagnostic methods to identify asymptomatic infections and detect atypical clinical manifestations during follow-up (7, 22). Molina et al. (21) demonstrated that HIV-infected individuals with asymptomatic *Leishmania* infection may transmit the parasite to sand flies, thereby contributing to the maintenance of VL transmission in endemic areas like the Mediterranean (23). To the best of our knowledge, few longitudinal studies have focused exclusively on the outcomes of asymptomatic coinfected patients. This prospective study (2017–2023) aimed to assess the clinical outcomes of asymptomatic *Leishmania*-HIV coinfected patients and *Leishmania-*negative PLWH in Northeast Brazil.

## 2 Materials and methods

### 2.1 Study design and samples

**Figure 1:**
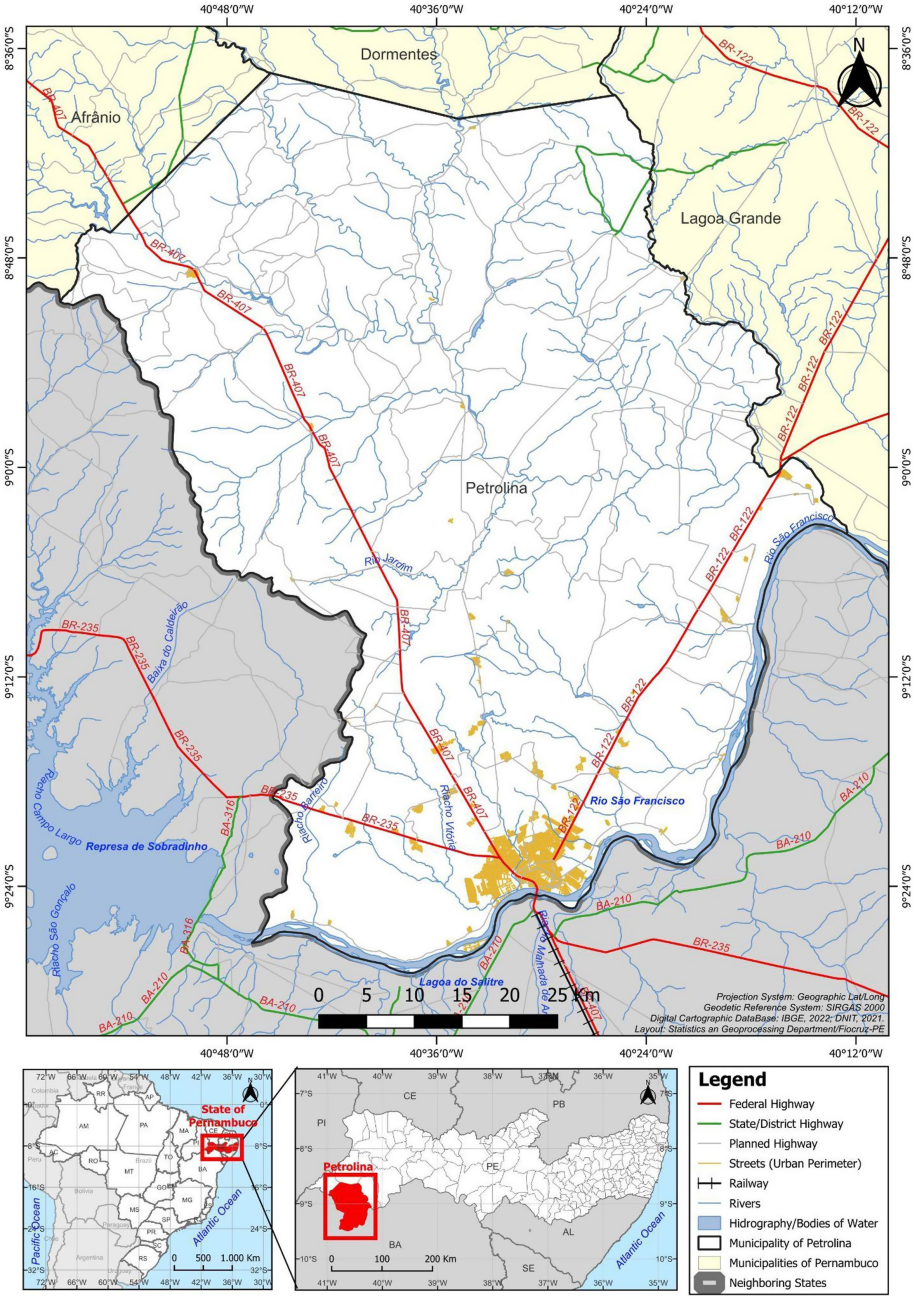
Location of the municipality of Petrolina in the state of Pernambuco, Brazil. Statistics and Geoprocessing Department, Instituto Aggeu Magalhães, Fundação Oswaldo Cruz (IAM/FIOCRUZ), 2025.

This was a prospective cohort study conducted from January 2017 to September 2023. We followed a cohort of PLWH, including those with asymptomatic *Leishmania*-HIV coinfection, in the city of Petrolina, State of Pernambuco, Northeast Brazil (7) (Figure 1).

The cohort was established based on a 2017 prevalence study that screened 483 PLWH, which identified 44 individuals as having asymptomatic *Leishmania*-HIV coinfection (7). Inclusion criteria at that time were PLWH 18 years or older who were being treated at the local HIV care service and were on regular Antiretroviral Therapy (ART). Exclusion criteria included current VL treatment, clinical symptoms of VL, or irregular ART use (7).

From 2017 to 2023, longitudinal clinical data were collected from medical records, specifically focusing on incident symptomatic VL, relapse, cause of death, treatment abandonment, and transfer of care. In 2023, the original participants were invited for retesting for *Leishmania*. A total of 29 asymptomatic *Leishmania*-HIV coinfected patients and 51 *Leishmania-*negative PLWH from the original cohort were successfully retested.

### 2.2 Cases definitions

- Asymptomatic *Leishmania*-HIV coinfection: An HIV-positive individual with at least one positive serological or molecular test for VL, but without clinical symptoms (7).
- Symptomatic *Leishmania*-HIV coinfection: An HIV-positive patient with laboratory confirmation of VL (parasitological, serological, or molecular test) and clinical symptoms (e.g., fever ≥ 2 weeks, hepatomegaly, splenomegaly, cough, diarrhea, dyspnea, bleeding, weight loss, or mucous pallor) (24).
- Relapse: Recurrence or worsening of one or more clinical signs (e.g., fever, cytopenia, and hepatosplenomegaly) within 12 months after clinical cure (24).
- *Leishmania-*negative PLWH: HIV-positive individuals testing negative for *Leishmania* markers (24).
- VL-related death: A death in a PLWH with VL symptoms and/or positive *Leishmania* tests where the cause of death was attributed to VL (24).
- Abandonment (loss to follow-up): Patients who failed to attend health services for over 3 months after starting treatment or missed appointments for over 6 months, including medication discontinuation or the breakdown of the patient-healthcare provider relationship (24).
- Transfer of care: Patients unable to continue attending the health center due to relocation (e.g., moving to a different city or state) (24).
- Regular ART use: Continuous antiretroviral therapy for at least 6 months (24).
- Seroreversion (or Reversion to negative): A change from positive to negative *Leishmania* markers in PLWH (25).

### 2.3 Clinical procedures and laboratory analysis

#### 2.3.1 Baseline (2017) and follow-up (2017–2023) data collection

In 2017, PLWH participants underwent interviews, physical examinations, and peripheral venous blood and urine samples were collected for *Leishmania* testing (7). From 2017 to 2023, medical records were reviewed for symptomatic VL, relapses, causes of death, abandonment, and transfers of care.

#### 2.3.2 Retesting procedures (2023)

In 2023, participants were re-evaluated with interviews, physical examinations, and peripheral blood collection. *Leishmania* testing included: Enzyme-Linked Immunosorbent Assay (ELISA)-rK39, the LSH Ab ECO rapid immunochromatographic test (ICT), the Direct Agglutination Test (DAT), and Polymerase Chain Reaction (PCR).

#### 2.3.3 Serological and immunological tests

For the ELISA-rK39 test, we used the recombinant lipoprotein antigen rK39 (Rekom Biotech, Granada, Spain). The assays were performed according to the protocol described by Pedras et al. (26) with minor adaptations. The LSH Ab ECO test (Eco Diagnóstica, Nova Lima, MG, Brasil) (27), which uses rK39 recombinant antigen, was executed the manufacturer’s instructions. For the DAT, we utilized a kit from the Institute of Tropical Medicine Antwerp (ITM-A), Belgium, following the instruction manual. Titers of ≥ 1:1600 were considered positive (28, 29).

#### 2.3.4 Molecular analysis (PCR)

For PCR, we targeted the kinetoplast DNA (kDNA) of *Leishmania* species. The primers used were 150 (5′-GGG(G/T)AGGGGCGTTCT(C/G)CGAA3’) and 152 (5′-(C/G)(C/G)(C/G)(A/T)CTAT(A/T)TTACACCAACCCC-3′). These primers amplify a 120 bp fragment common to all *Leishmania* species. PCR conditions were described by Souza et al. (30). To confirm the species as *L. infantum*, we used the following primers, which amplify a 230 bp product, according to Gualda et al. (31): RLC2 (5’-GGGAAATTGGCCTCCCTGAG-3′) and FLC2 (5’-GTCAGTGTCGGAAACTAATCCGC-3′). Results were analyzed by electrophoresis on 1.5% agarose gels stained with ethidium bromide and visualized under ultraviolet light.

#### 2.3.5 Additional laboratory data

Data on blood count, biochemistry, CD4 + T cell count, and HIV viral load were obtained from medical records.

### 2.4 Ethics statement

The study protocol was approved by the Ethics Committee of the Instituto Aggeu Magalhães – Oswaldo Cruz Foundation in Pernambuco (approval number 33320920.3.0000.5190). All participants were informed about the study procedures and provided Written Informed Consent (WIC).

### 2.5 Data analysis

Data were entered and stored in spreadsheets using Microsoft Excel Professional Plus 2016 (Microsoft Corp., Redmond, WA, United States). Statistical analysis was performed using R software, version 4.2.2.

The incidence rate of symptomatic VL was calculated by dividing the number of incident symptomatic VL cases by the total person-time of follow-up among the asymptomatic *Leishmania*-HIV coinfected patients and the *Leishmania-*negative PLWH groups at baseline.

We also calculated the rate of seroreversion (reversion to negative *Leishmania* markers) among previously diagnosed *Leishmania*-HIV coinfected patients. This rate was defined as the number of patients with all negative *Leishmania* tests at the end of follow-up, divided by the total number of asymptomatic *Leishmania-*HIV coinfected individuals, and presented per 100 people (28).

For descriptive statistics, frequencies with 95% Confidence Intervals (CIs) were calculated. Measures of central tendency were reported as medians and Interquartile Ranges (IQRs) or means and Standard Deviations (SDs), depending on the data distribution. A bivariate analysis was performed to assess the relationship between variables and the outcome. Categorical variables were compared using the Odds Ratio (OR), estimated via the Wald statistic. The significance level was set at 5%, and tests yielding a *p*-value ≤ 0.05 were considered statistically significant.

## 3 Results

During the follow-up period (2017–2023), participant retention differed between the two groups. Out of the 44 participants in the asymptomatic *Leishmania*-HIV coinfected group, 32 remained in the study by 2023. Losses were attributed to: 7 loss to follow-up (abandonment), 1 death due to VL, and 4 transfers of care to other healthcare services. In the PLWH group (initial *n* = 439), 368 remained in 2023. Losses in this group were due to: 45 transfers of care, 6 loss to follow-up, and 20 deaths from causes unrelated to VL.

**Figure 2:**
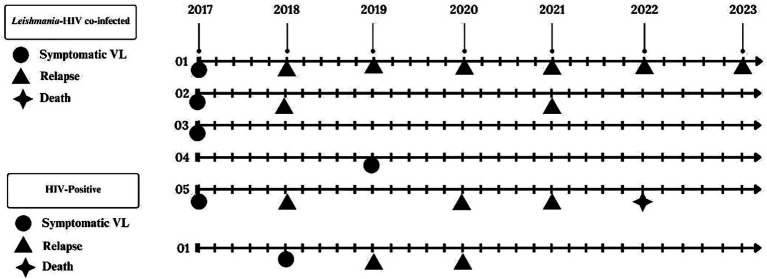
Participant flow during the prospective follow-up (2017–2023) of *Leishmania*-negative people living with HIV (PLWH) and asymptomatic *Leishmania*–HIV coinfected patients in Northeast Brazil.

By 2023, 29 participants from the *Leishmania*-HIV coinfected group and 51 from the PLWH group were successfully retested. Three individuals from the coinfected group and one from the PLWH group refused to participate in the retesting procedures (Figure 2).

Of the 29 retested asymptomatic *Leishmania*-HIV coinfected patients, five remained positive by one or more *Leishmania* tests during the follow-up. In the PLWH group (*n* = 51), two individuals seroconverted for *Leishmania* infection, testing positive by the ELISA-rK39 test (Table 1).

**Table 1:** Summary of *Leishmania* diagnostic test results among retested participants in Northeast Brazil (2017–2023).

*Leishmania*-HIV coinfected group (2017–2022 *n* = 44 and 2023 *n* = 29)							
VL tests^a^	2017	2018	2019	2020	2021	2022	2023
Bone marrow aspirate	3	4	1	2	3	1	1
ELISA-rK39	12	–	–	–	–	–	2
rK39-ICT	5	–	–	–	–	–	0
DAT	17	–	–	–	–	–	0
PCR kDNA	11	–	–	–	–	–	0
DAT and rK39 ICT	1	–	–	–	–	–	0
ELISA-rK39 and DAT	0	–	–	–	–	–	1
ELISA-rK39 + DAT + rK39-ICT	0	–	–	–	–	–	1
ELISA-rK39 + DAT + rK39-ICT + PCR kDNA	0	–	–	–	–	–	1
Total (at last one positive test)	**44**						**5**

PLWH group (2017–2022 *n* = 439 and 2023 *n* = 51)							
VL tests^a^	2017	2018	2019	2020	2021	2022	2023
Bone marrow aspirate	–	1	1	1	–	–	–
ELISA-rK39	0	–	–	–	–	–	2
rK39-ICT	0	–	–	–	–	–	0
DAT	0	–	–	–	–	–	0
PCR kDNA	0	–	–	–	–	–	0
Total (at last one positive test)	**0**						**2**

a VL tests were conducted in 2017 and 2023. Bold values indicate the total number of participants with at least one positive VL test result in each respective year.

**Figure 3:**
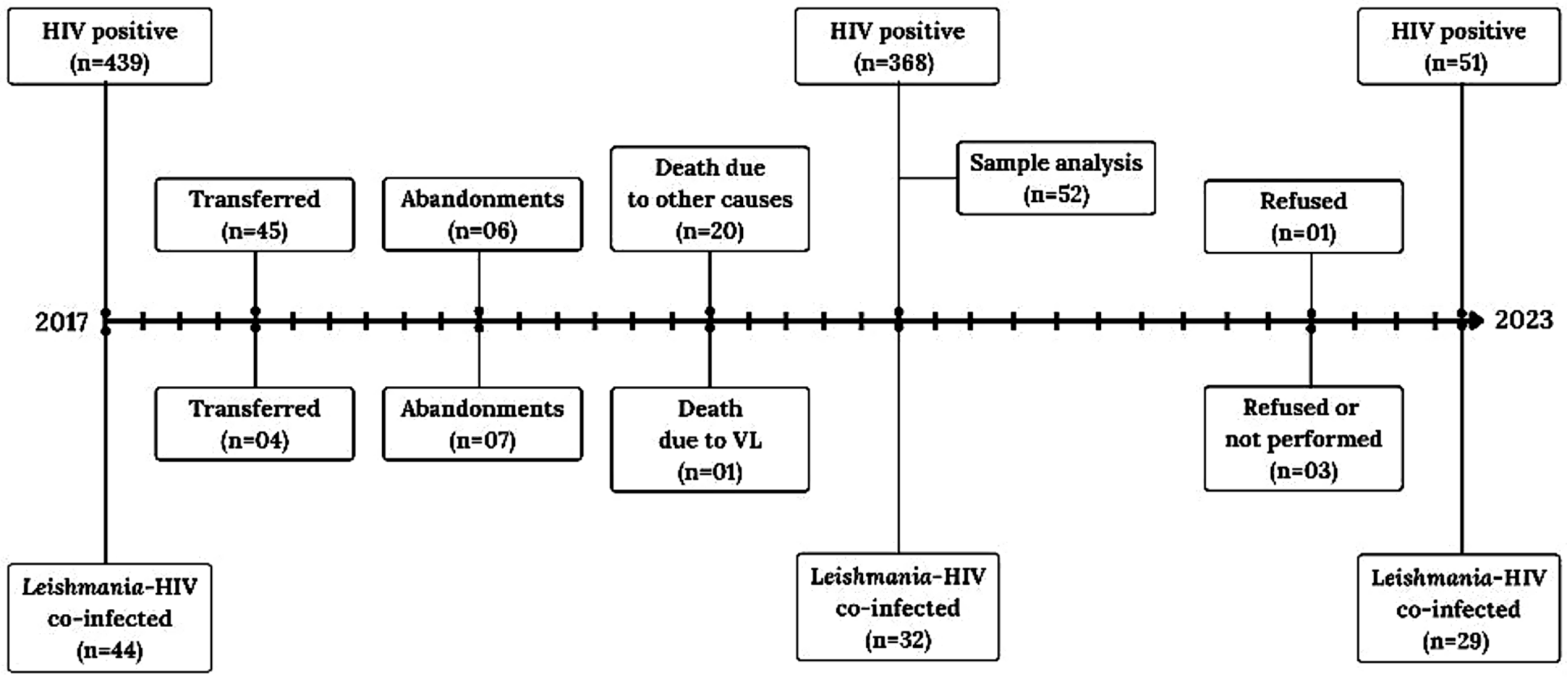
Incident VL, relapse, and mortality during prospective follow-up (2017–2023) of *Leishmania*-HIV coinfection in Northeast Brazil.

Clinical outcomes are summarized in Figure 3. A total of six participants developed symptomatic VL: five from the asymptomatic *Leishmania*-HIV coinfected group and one from the *Leishmania*-negative PLWH group. Three patients experienced VL relapse: one was from the PLWH group (the individual who seroconverted for *Leishmania* infection) and two were from the asymptomatic coinfected group. Regarding the relapse cases, two participants experienced two episodes each, while one individual suffered six episodes (one per year).

Comparison between groups revealed that both cohorts predominantly resided in urban areas; however, a significantly higher proportion of *Leishmania*-HIV coinfected participants lived in rural areas compared to *Leishmania*-negative PLWH (OR 7.20; 95% CI 1.76–29.4; *p* = 0.004). Asymptomatic coinfected patients were significantly more likely to report weight loss (OR 3.77; 95% CI 1.2–11.88; *p* = 0.025). In contrast, splenomegaly was only observed in the *Leishmania*-negative PLWH group (Tables 2, 3).

**Table 2:** Comparison of epidemiological features between *Leishmania*-negative PLWH and asymptomatic *Leishmania*-HIV coinfected participants retested in 2023 in Northeast Brazil.

Variables	PLWH		*Leishmania*-HIV coinfected		OR		*p*-value
	*n* = 51	%	*n* = 29	%	OR	95% CI	
Gender							
Male	33	64.7	15	51.7			
Female	18	35.3	14	48.3	1.71	0.68–4.33	0.268
Age (years)							
18–28	3	5.9	2	6.9			
29–39	12	23.5	6	20.7	0.75	0.10–5.77	0.794
40–49	7	13.7	8	27.6	2.29	0.22–13.41	0.652
>50 or more	29	56.9	13	44.8	0.39	0.10–4.52	0.695
Years of schooling							
0–8	22	43.1	14	48.3			
9–11	4	7.8	3	10.3	1.18	0.23–6.08	0.846
12 or more	25	49.0	12	41.4	0.64	0.29–1.97	0.576
Area							
Urban	48	94.1	20	69.0			
Rural	3	5.9	9	31.0	7.20	1.76–29.40	0.004
Dogs at home							
No	29	56.9	14	48.3			
Yes	22	43.1	15	51.7	1.41	0.57–3.53	0.471
VL in patients’ household							
No	50	98	28	96.6			
Yes	1	2.0	1	3.4	1.79	0.11–29.67	0.725
VL in patients’ neighborhood							
No	44	86.3	27	93.1			
Yes	7	13.7	2	6.9	0.47	0.09–2.41	0.388
Resident in an endemic area (last 2 years)							
No	12	23.5	6	20.7			
Yes	39	76.5	23	79.3	1.18	0.39–3.57	0.788

*n*, number of patients in the group.

**Table 3:** Comparison of clinical and laboratory parameters between *Leishmania*-negative PLWH and asymptomatic *Leishmania*-HIV coinfected participants retested in 2023 in Northeast Brazil.

Variables	PLWH		*Leishmania*-HIV co-infected		OR		*p*-value
	*n* = 51	%	*n* = 29	%	OR	95% CI	
Presence of fever							
No	43	84.3	24	82.8			
Yes	8	15.7	5	17.2	1.12	0.33–3.81	0.850
Presence of hepatomegaly							
No	43	84.3	27	93.1			
Yes	2	3.9	2	6.9	1.59	0.21–11.98	0.676
Presence of splenomegaly							
No	43	84.3	27	93.1			
Yes	–	–	2	6.9	–	–	–
Presence of hepatosplenomegaly							
No	43	84.3	27	93.1			
Yes	2	3.9	2	6.9	1.59	0.21–11.98	0.676
Presence of cough							
No	43	84.3	26	89.7			
Yes	13	25.5	3	10.3	0.38	0.10–1.47	0.161
Presence of diarrhea							
No	43	84.3	27	93.1			
Yes	7	13.7	2	6.9	0.46	0.09–2.35	0.373
Presence of dyspnea							
No	43	84.3	25	86.2			
Yes	8	15.7	4	13.8	0.86	0.23–3.15	0.843
Presence of bleeding							
No	43	84.3	24	82.8			
Yes	6	11.8	5	17.2	1.49	0.41–5.41	0.550
Presence of weight loss							
No	43	84.3	19	65.5			
Yes	6	11.8	10	34.5	3.77	1.20–11.88	0.025
Presence of mucous pale							
No	43	84.3	27	93.1			
Yes	3	5.9	2	6.9	1.06	0.17–6.77	0.936
Patients with Diabetes mellitus							
No	43	84.3	26	89.7			
Yes	11	21.6	3	10.3	0.45	0.12–1.77	0.264
Patients with hypertension							
No	43	84.3	24	82.8			
Yes	15	29.4	5	17.2	0.60	0.19–1.85	0.387
Patients with obesity							
No	43	84.3	27	93.1			
Yes	1	2.0	2	6.9	3.19	0.28–36.85	0.405
Patients with cardiovascular disease							
No	43	84.3	29	100.0			
Yes	3	5.9	–	–	–	–	–
Patients with neoplasm							
No	43	84.3	28	96.6			
Yes	1	2.0	1	3.4	1.54	0.09–25.57	0.795
Patients with respiratory disease							
No	43	84.3	27	93.1			
Yes	3	5.9	2	6.9	1.06	0.17–6.77	0.936
TCD4 + count (cells/mm^3^)							
<200	5	9.8	6	20.7			
200–349	9	17.6	4	13.8	0.37	0.07–1.97	0.279
>350	37	72.5	19	65.5	0.43	0.12–1.59	0.223
HIV viral load (copies/mL)							
Undetectable (<50)	39	76.5	21	72.4			
50–100,000	9	17.6	6	20.7	1.24	0.39–3.95	0.721
>100,000	3	5.9	2	6.9	1.24	0.19–8.00	0.819
In use of ART							
No	3	5.9	–	–	–	–	–
Yes	48	94.1	29	100.0	–	–	–
General Laboratory – Median (IQR) to the variables below							
White blood cells (cells/mm^3^)	5600 (4600–7010)			5250 (4302.5–6407.5)			0.360
Neutrophils	3086 (2189.3–3860.5)			3096 (2239–3634)			0.931
Lymphocytes	1999.5 (1722.8–2689.5)			1875 (1388.5–2587)			0.224
Hemoglobin (g/dL)	13.6 (12.5–15.1)			14.2 (13.2–14.7)			0.361
Platelets (cells/mm^3^)	237 (197.5–274.5)			217.5 (176.5–269)			0.187
HTC	42.3 (37.5–46.1)			41.8 (39.3–43.6)			1.000
AST (U/L)	26 (19.3–32.5)			25 (22.8–37)			0.413
ALT (U/L)	24.5 (17.3–36.8)			25.5 (18.5–36.3)			0.632
Creatinine (mg/dL)	0.9 (0.8–1.1)			0.9 (0.7–1.1)			0.762

*n*, number of patients; ART, antiretroviral therapy; AST, aspartate aminotransferase; ALT, alanine aminotransferase.

Table 4 summarizes the incidence of symptomatic VL. During follow-up, the cumulative incidence was 0.17 (5/29) in the asymptomatic *Leishmania*-HIV group, compared with 0.02 (1/51) in the *Leishmania*-negative PLWH group. The comparison showed that the coinfected group had a significantly higher cumulative incidence of VL (OR 10.4, 95% CI 1.2–94.2, *p* = 0.023). Among the 29 retested coinfected participants, 22 seroreverted to negative *Leishmania* markers, resulting in a seroreversion rate of 75.9 per 100 people over the 2017–2023 follow-up period.

**Table 4:** Comparison of VL cumulative incidence and risk of symptomatic disease between asymptomatic *Leishmania*–HIV coinfection and *Leishmania*-negative PLWH (2017–2023).

Groups	Symptomatic infection				OR	95% CI	*p*-value
	No		Yes				
	*n* = 74	%	*n* = 6	%			
PLWH	50	98.0	1	3.4			
*Leishmania*-HIV co-infected	24	47.1	5	17.2	10.4	1.2–94.2	0.023

*n*, number of patients.

## 4 Discussion

This study reports the outcomes of a six-year prospective follow-up among two outpatient groups: asymptomatic *Leishmania*-HIV coinfected individuals and *Leishmania*-negative PLWH. During the study period, five asymptomatic coinfected participants progressed to symptomatic VL, compared to only one participant in the PLWH group. Statistically, the coinfected group had a significantly higher cumulative incidence of VL (OR 10.4, 95% CI 1.2–94.2, *p* = 0.023). These findings strongly reinforce existing evidence (7, 28) that PLWH living in VL-endemic areas are at risk of acquiring *Leishmania* infection and that asymptomatic coinfection is a major risk factor for progression to symptomatic VL.

Few national-level studies in Brazil have addressed the prevalence of *Leishmania*-HIV coinfection (32, 33), consistently highlighted endemic regions for VL, including the Distrito Federal (34), Ceará (6), Maranhão (35), Mato Grosso (36), Mato Grosso do Sul (37), Minas Gerais (38, 39), Piauí (12), Rio Grande do Norte (40), Sergipe (41), and Tocantins (42). Specifically in Pernambuco, an endemic state for VL, Cavalcanti et al. (16) reported 10 symptomatic *Leishmania*-HIV coinfection cases among hospitalized PLWH, and Guedes et al. (43) identified a 16.9% prevalence in Recife. In Petrolina (the setting of our study), Diniz et al. (15) reported a 16.8% (107/181) of coinfection using secondary data. More recently, the baseline of the current study reported a 9.1% (44/483) prevalence of asymptomatic *Leishmania-*HIV coinfection in the same region (7). Crucially, this is the first prospective study in Brazil designed to follow and evaluate the long-term outcomes of this specific population.

The diagnosis of asymptomatic *Leishmania* infection in VL-endemic areas is critical for effective PLWH surveillance and enabling timely intervention should disease progression occur. The challenges in diagnosing asymptomatic *Leishmania* infection are underscored by the current lack of diagnostic consensus, resulting in the use of various techniques, including parasitological (culture and microscopy), molecular (cPCR, qPCR, LAMP), serological (ELISA, ICT, DAT), and cellular *Leishmania* skin test (LST), whole blood assay (WBA), cell proliferation assay (CPA) tests (44). In our study, we employed a combination of serological (ELISA, DAT, ICT) and molecular (cPCR) tests across both evaluation periods (2017 and 2023).

Our findings underscore the utility of serological tests for screening and surveillance asymptomatic coinfected individuals, consistent with previous studies (7, 25). However, the LSH Ab ECO rapid test showed a low detection rate, consistent with reports of limited sensitivity for detecting *Leishmania* infection in PLWH residing in *L. infantum*-endemic regions (45, 46). In contrast, the ELISA-rK39 test demonstrated the highest positivity rate in the coinfected group (47, 48), further supporting the use of combined diagnostic approaches for detecting asymptomatic infection.

The Brazilian Ministry of Health recommends performing *Leishmania* serology at the first post-HIV diagnosis appointment for individuals in endemic areas; nevertheless, this is not yet widely implemented in routine clinical practice. Despite implementation challenge, *Leishmania* testing for PLWH in VL-endemic areas is a core component of the Brazilian Ministry of Health’s control and elimination policies, which could significantly improve treatment and patient outcomes (15, 49, 50). Asymptomatic coinfection remains poorly understood (6, 51). Critically, these individuals may act as reservoirs of infection, potentially contributing to *Leishmania* transmission due to high parasitic loads, as noted by Molina et al. (21).

Clinical findings revealed a higher frequency of reported weight loss in asymptomatic *Leishmania*-HIV coinfected group compared to *Leishmania*-negative PLWH. This is notable, as other studies from Brazil (39, 43, 51, 52) and Ethiopia (53) have typically reported weight loss in symptomatic coinfected patients. HIV-induced immunosuppression weakens the body’s defenses against intracellular parasites like *Leishmania* species. Furthermore, *Leishmania* can promote the intracellular replication of HIV, which accelerates the clinical progression of HIV infection and increases symptoms (42). Since weight loss is common in both VL and advanced HIV infection, its higher frequency in our asymptomatic coinfected patients is particularly concerning.

Consistent with Cesse et al. (54), who demonstrated *Leishmania* transmission in urban areas of Petrolina, our study also identifies asymptomatic *Leishmania*-HIV coinfection in these settings. This urban finding aligns with the documented epidemiological shift in states like Rio Grande do Norte (55) and Piauí (56), where migration from rural to urban areas has led to *Leishmania* infection in urban or peri-urban populations. This pattern is not unique to Brazil; studies in Morocco (57, 58), Italy (10), Iran (59), Mexico (60), and Colombia (61) have similarly reported a higher proportion of symptomatic *Leishmania-*HIV coinfected patients in urban areas.

Our study showed that the majority of participants in both groups maintained a CD4^+^ T-cell count of > 350 cells/mm^3^ (*p* = 0.223), consistent with reports from PLWH in Rio Grande do Norte, Brazil (62). However, when observing patients with advanced immunosuppression (CD4^+^ < 200 cells/mm^3^), a higher proportion was noted in the *Leishmania*-HIV coinfected group (20.7%) than in the *Leishmania*-negative PLWH group (9.8%). The lower CD4^+^ T-cell count observed in *Leishmania*-HIV coinfected patients may be explained by reduced IFN-*γ* production in response to *Leishmania* antigens, contributing to atypical parasitic dissemination, frequent relapses, and treatment failure (63).

Our findings identified a non-significant trend toward a higher prevalence of diabetes mellitus, cardiovascular diseases, and hypertension among coinfected individuals compared to the PLWH (*p* > 0.05). The literature has already associated these comorbidities with PLWH in Brazil (64, 65) and Spain (66). For instance, Pandey et al. (67) reported a case of a 50-year-old Indian male coinfected with *Leishmania* and HIV who also presented with Parkinsonism, diabetes mellitus, and hyperuricemia. Comorbidities associated with aging, including diabetes, high blood pressure, and cardiovascular diseases, can further compromise the immune system. This increased susceptibility to infection in older adults, may exacerbate the vulnerability of those with HIV and *Leishmania*-HIV coinfection.

We found 22 cases of seroreversion to negative *Leishmania* tests among asymptomatic coinfected individuals, resulting in a seroreversion rate of 75.9 per 100 individuals over the 2017–2023 follow-up period. In comparison, a study from northwest Ethiopia (28) found that 16 of 49 individuals with prevalent infection at baseline seroreverted during follow-up (2015–2016), yielding a rate of 48.5 per 100 persons-year. The differences in these rates can be partially attributed to the varying follow-up durations between the two studies.

In the present study, the cumulative incidence of symptomatic VL was significantly higher among asymptomatic *Leishmania*-HIV coinfected patients compared to the *Leishmania*-negative PLWH. High frequencies of symptomatic VL, recurrence, and increased mortality, along with a higher rate of relapses and treatment failures, are typically observed among *Leishmania*-HIV coinfected patients (18, 68). During our follow-up, the risk of progression to symptomatic VL was 10.4 times higher in asymptomatic coinfected patients compared to PLWH living in an VL-endemic area from Northeast Brazil (OR 10.4; 95% CI 1.2–94.2; *p* = 0.023). Overall, three patients experienced relapse: one was the individual from the *Leishmania*-negative PLWH group who seroconverted, and two were from the asymptomatic *Leishmania*-HIV coinfected group.

Additionally, one VL-related death occurred in our study, originating from the asymptomatic *Leishmania*-HIV coinfected group. In a previous national analysis of 18,501 human VL cases in Brazil (2007–2011), researchers identified risk factors associated with death. The study reported that, at the time of clinical suspicion, death was predicted by the presence of leishmaniasis in PLWH patients (33). HIV seropositivity was the most significant predictor of death among individuals treated for VL, with HIV-infected individuals being four times more likely to die than HIV-negative individuals (69). This high mortality risk is compounded by the fact that, in Brazil, VL cases are often investigated only after severe symptoms are present, which hinders the timely diagnosis of asymptomatic or mild *Leishmania* infection (11).

Limitations of our study included the difficulty in performing repeated laboratory tests for *Leishmani*a throughout the follow-up, given that patients were primarily monitored by local health services physicians. To mitigate this, information on clinical signs and symptoms was diligently obtained from medical records. Furthermore, the COVID-19 pandemic occurred during the study period, which resulted in changes to healthcare routines that may have affected the monitoring of HIV cases, including ART adherence. A critical limitation is the lack of standardized diagnostic and long-term follow-up protocols for asymptomatic *Leishmania*-HIV coinfection, which complicates care in HIV outpatient clinics in VL-endemic regions. Therefore, future prospective studies, building upon our findings, are essential to support the development of such protocols.

## 5 Conclusion

Our six-year prospective study provides critical insights into the long-term outcomes of *Leishmania*-HIV coinfected patients in VL-endemic areas. It demonstrates that asymptomatic coinfected individuals have a significantly elevated risk of progression to symptomatic VL and subsequent recurrence compared to *Leishmania*-negative PLWH. Research focusing on asymptomatic *Leishmania* carriers is crucial for evidence-based public health policymaking in endemic regions, directly contributing to the control and prevention of adverse outcomes. Our findings emphasize the critical need for routine, long-term surveillance and follow-up care for individuals with asymptomatic *Leishmania*-HIV coinfection to prevent disease progression and mitigate life-threatening complications.

## Data Availability

The original contributions presented in the study are included in the article/supplementary material, further inquiries can be directed to the corresponding authors.
